# Evaluation of Circulation, Γ, as a quantifying metric in 4D flow MRI

**DOI:** 10.1186/1532-429X-15-S1-E36

**Published:** 2013-01-30

**Authors:** Aaron T Hess, Malenka M Bissell, Steffan J Glaze, Alex Pitcher, Saul Myerson, Stefan Neubauer, Matthew D Robson

**Affiliations:** 1 OCMR, Cardiovascular Medicine, University of Oxford, Oxford, UK

## Background

4D flow MRI provide a wonderfully rich set of data for diagnosis and understanding of cardiac disease. Interpretation of this wealth of data can be challenging and it may be desirable to simplify it to make analysis manageable. We have observed interesting rotational behavior of blood in the aorta in a number of cardiac diseases. Circulation (Γ) is a metric used in fluid dynamics to quantify the rotational components of flow. This metric has not been reported before for 4D flow MRI and may provide a measurement that can be used diagnostically. The purpose of this work is to define the normal range of Γ in the ascending aorta and investigate its reproducibility.

## Methods

Circumferential circulation is calculated as the integral of vorticity (ω) with respect to area within a transverse plane through the aorta, Γ= ∫∫ω **dS** (Stokes' theorem). Γ is a 3D vector, whose through plane component, Γ_T_, depicts the within plane (circumferential) rotation. Γ_T_ was calculated in the ascending aorta at the level of the pulmonary artery, both at peak systole and during mean systole (-80 to 120 ms about peak). 35 healthy volunteers were assessed, mean age 36.7 ±17.6 years, range from 8 to 74 years old, 23 male and 12 female. Three volunteers were rescanned to assess repeatability. 4D Flow [1] was analyzed in EnSight (CEI, Apex, NC, USA) to calculate vorticity maps. A slice through the lumen was segmented to include only the blood pool, and a discrete integral with respect to voxel cross-sectional area was calculated in Matlab (Natick, MA, USA).

## Results

**Figure 1:**
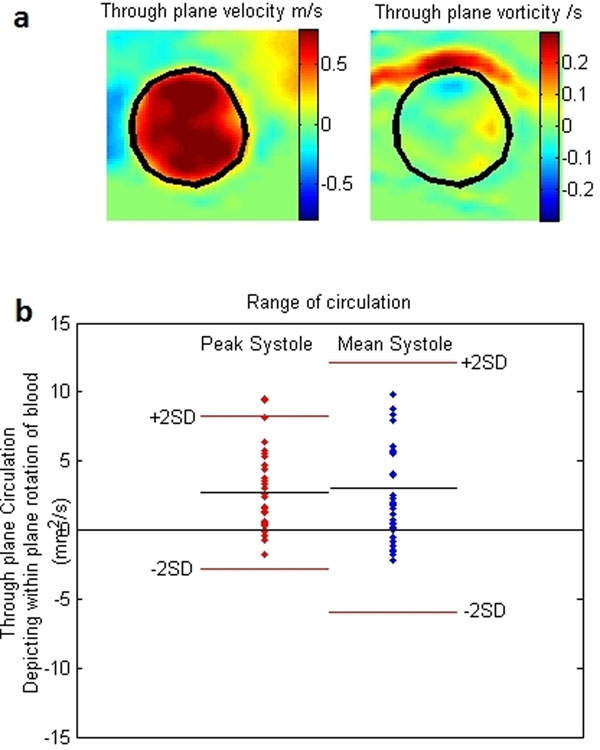
a) Segmentation of aorta on velocity map and vorticity map. b) Range of peak and mean systolic circumferential circulation in the aorta at the level of the pulmonary artery.

Figure 1 demonstrates the segmentation on a velocity and vorticity map. In our group of 35 volunteers mean ±SD of Γ_T_ was 2.71 ±2.78 mm^2^/s and 3.07 ±4.51 mm^2^/s for peak and mean systole respectively. Our three repeated scans have differences and means shown in table 1. They show that the intra-subject variation is less than the inter-subject variation, this supports the reproducibility of the method. Figure 2 demonstrates the range of circulations calculated. In patients with heart valve disease we have observed values of Γ_T_ at peak as high as 67 and -80 mm^2^/s and over mean systole of 62 and -62 mm^2^/s.

**Table 1:** Mean and difference of repeated scans

Subject/Method	Difference mm^2^/s	Mean mm^2^/s
1/peak systole	1.81	1.50
2/peak systole	-1.02	8.66
3/peak systole	0.91	2.90
1/mean systole	-0.50	0.44
2/mean systole	0.93	15.44
3/mean systole	2.04	1.11

## Conclusions

Circulation (Γ_T_) has been found to be straightforward to calculate and a robust means of quantitating the rotational flow characteristic in the ascending aorta. The normal range of values calculated here are found to be positive and low compared to those to those seen in disease which can be high or negative, which suggests that this approach may have high clinical sensitivity.

## Funding

The MRC (UK), and the British Heart Foundation
